# Antibiotic Use in Organic and Non-organic Swedish Dairy Farms: A Comparison of Three Recording Methods

**DOI:** 10.3389/fvets.2020.568881

**Published:** 2020-10-30

**Authors:** Gabriela Olmos Antillón, Karin Sjöström, Nils Fall, Susanna Sternberg Lewerin, Ulf Emanuelson

**Affiliations:** ^1^Department of Clinical Sciences, Faculty of Veterinary Medicine and Animal Science, Swedish University of Agricultural Sciences, Uppsala, Sweden; ^2^Department of Biomedical Sciences and Veterinary Public Health, Faculty of Veterinary Medicine and Animal Science, Swedish University of Agricultural Sciences, Uppsala, Sweden

**Keywords:** BIN method, national surveillance systems, farm level, DDD_*vet*_ metric, DCD_*vet*_ metric, AMU

## Abstract

Biases of antimicrobial use (AMU) reporting systems pose a challenge to monitoring of AMU. Our study aimed to cross-compare three data sources of AMU in Swedish dairy herds to provide an account of the validity of AMU reports. We studied AMU differences between two production systems, to investigate how the reporting system affected this comparison. On-farm quantification of AMU via a manual collection of empty drug containers (BIN) took place in organic (*n* = 30) and conventional (*n* = 30) dairy herds during two periods between February 2016 and March 2017. A data extract mirroring these periods was obtained from two linked datasets that contain AMU data as reported by the prescribing veterinarians. These included data from the Swedish Board of Agriculture system (SBA) and Växa milk recording system (VXA). Using the European Medicines Agency technical units, the total number of defined daily doses (DDD_vet_), and defined course doses (DCD_vet_) per animal/year were calculated for each herd/period/dataset. Descriptive statistics and Bland–Altman plots were used to evaluate the agreement and systematic bias between the datasets. Mixed models for repeated measures were used to assess AMU differences between production systems. We found consistent numerical differences for the calculated AMU metrics, with BIN presenting higher usage compared to the SBA and VXA. This was driven by a disparity in intramammary tubes (IMt) which appear to be underreported in the national datasets. A statistically significant interaction (BIN dataset) between the production system and drug administration form was found, where AMU for injectable and lactating cow IMt drug forms differed by the production system, but no difference was found for dry-cow IMt. We conclude that calculating AMU using DDD_vet_ and DCD_vet_ metrics at a herd level based on Swedish national datasets is useful, with the caveat of IMt potentially being misrepresented. The BIN method offers an alternative to monitoring AMU, but scaling up requires considerations. The lower disease caseload in organic herds partly explains the lower AMU in particular drug forms. The fact that organic and conventional herds' had equally low AMU for dry-cow IMt, coupled with mismatches in IMt report across herds indicated an area of further research.

## Introduction

Antimicrobial resistance (AMR) is a global issue, and the current pattern and reduction in its harmful consequences to the biosphere's health (1, 2) require concerted actions from the human, animal, agricultural, and environmental sectors (3). A key measure for AMR mitigation is reducing antimicrobial use (AMU), especially “Critically Important Antimicrobials” (CIAs) for human health (4–6). The livestock industry is predicted to be responsible for 70% of the global AMU by 2030 (2, 7) and the possible relationship between AMU in animal production, and the development of AMR has been highlighted (8). Despite the association between AMR in livestock and humans, there is uncertainty about its magnitude (9–11).

Measuring AMU is fundamental for monitoring and reduction of AMU. Key indicators for understanding the patterns include (a) monitoring trends over time; (b) comparisons between different populations (e.g., types of production, species, or countries); (c) benchmarking; and (d) study of associations between AMU and AMR (12). To date, no standardised AMU measurement fulfils all these objectives. Thus, suitable measurement(s) must be determined based on a trade-off between the set goals and data at hand. Data resolution, comprehensiveness, and stability over time are important for the assessment of exposure and comparison of AMU within and between populations (12, 13).

All the practicalities around collection and reporting, regardless of the chosen resolution (e.g., animal, herd, or country level), have a high impact on AMU measurements. In turn, this affects the transparency and comparability of figures obtained. Despite this, only a few studies have addressed the qualitative and quantitative biases of AMU reporting systems (12, 14–16).

In Sweden, AMU in animals is only allowed on veterinary prescription, and veterinary drugs are sold exclusively through registered pharmacies. Sweden has had a leading role in the reduction of AMU, as well as the quantification and reporting of AMU statistics in animals and humans via the Swedres-Svarm reports (17). These reports are based on national sales data, which are not always the same as the amount prescribed or used in a country.

Although no recent effort has been made to evaluate AMU at herd level in Swedish dairy herds (18), the tools to do so are available through official records of veterinary treatments. The “Djursjukdata DAWA” is owned by the Swedish Board of Agriculture (in Swedish: Jordbruksverket). This is the oldest data collection system for veterinary treatments in Sweden, including antimicrobial use at herd level, initially compiled via paper records and launched at a national level in 1984. Currently, a computerised system covers all food-producing animals and horses, although 30% of the data is still fed to the system in paper format. Moreover, this system provides health information to Växa, the biggest dairy Levy group in Sweden that aims to monitor and improve the productivity, health and welfare of Swedish dairy cows (19). Växa's national database thus opens the window to understanding AMU in the context of detailed herd characteristics not available in the DAWA system.

Based on the EU rules for organic farming, dairy herds are expected to maintain a restrictive AMU (20). In practice, these rules limit the number of treatments per animal/year, and depending on the member state, might include the extension of drug withdrawal periods or the promotion of alternative medicines. Since conventional herds do not have to abide by such rules, it is often assumed that AMU would be higher in these herds. Although some studies have confirmed that organic herds have a lower AMU than conventional herds (5, 21–25), this research area remains unexplored in Sweden. The requirements may also impact the role of prescribing veterinarians under the two systems and, thus, also the AMU recordings. Hence, a comparison between AMU in organic and conventional dairy herds provides a case for AMU data evaluation.

This study aimed to provide a qualitative and quantitative assessment of how data source affects AMU reports, including treatment characteristics and characteristics of the population treated and at risk within a herd. Additionally, we wanted to quantify, describe, and compare the AMU in organic and conventional Swedish dairy herds and explore if AMU recording differs between the systems.

## Methodology

### Herd Enrolment

The study design adhered to the good scientific practice guidelines set out by Swedish legislation and did not include direct animal testing. Thus, no ethical evaluation or permit was required for its execution. Suitable farms were invited to participate through veterinarians, farmer's organisations and advisors. The farms were chosen to reflect the size and distribution of dairy herds in Sweden. Each organic and conventional farm was geographically matched. The overall aims of the study were explained to the farmers who, when they agreed to join, signed a form that approved the use of data from their herds for research purposes within the context of this study. A total of 60 farmers were enrolled in the project, 30 organic and 30 conventional dairy farms.

### Data Collection

**Figure 1:**
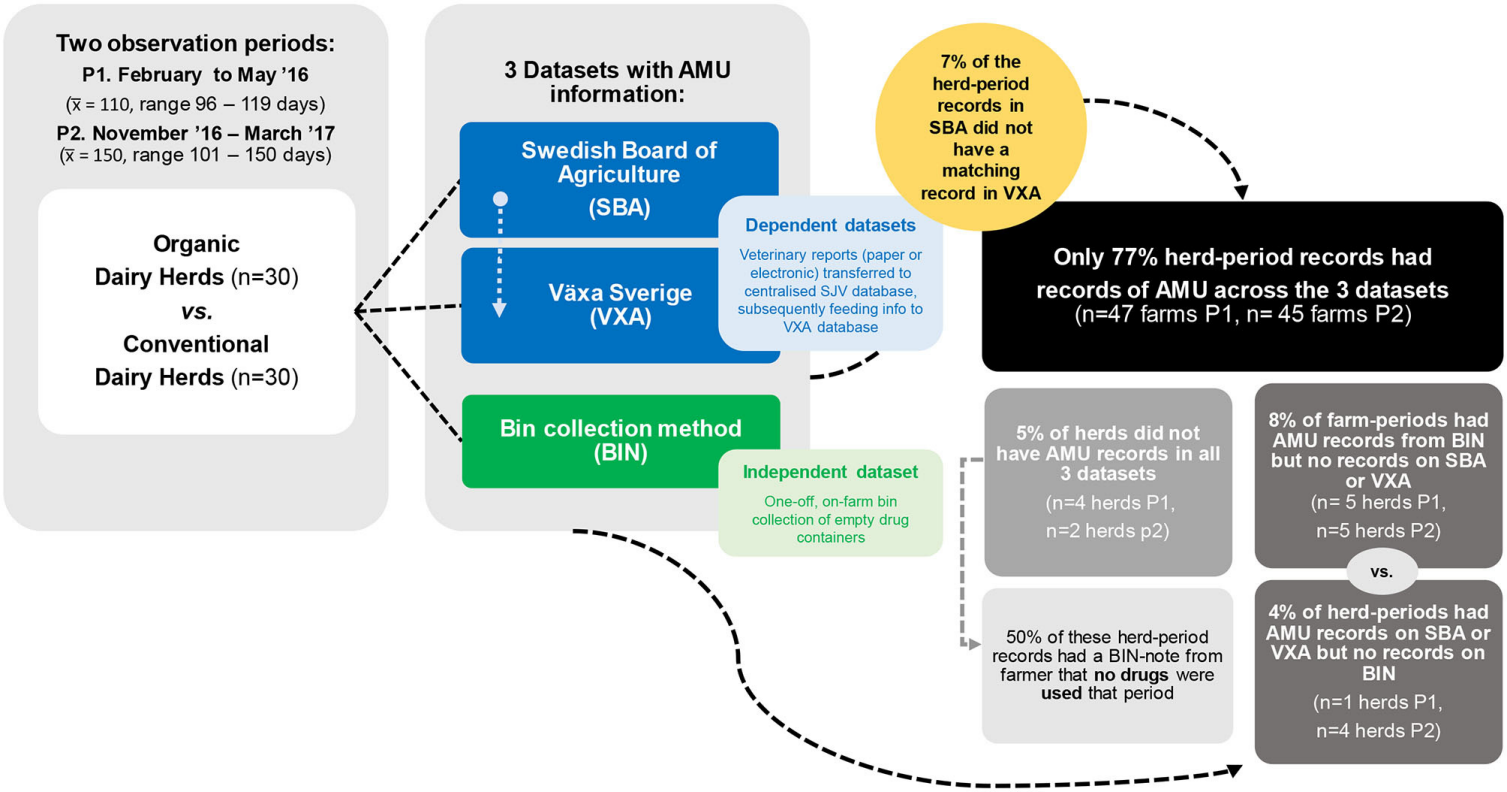
Flow diagram of methodology, dataset relationship, and data availability for antimicrobial use collection for a sample of 30 organic and 30 conventional Swedish dairy herds across two observation periods and as reported by three different datasets.

The AMU was assessed during two periods: February to May 2016 and November 2016 to March 2017 using three data sources (see Figure 1). Two data sources are based on antimicrobial prescription data [Växa Sverige (VXA) and Swedish Border of Agriculture (SBA)] while the third one [empty drug containers (BIN)] compiled data on actual AMU on farm.

#### On-Farm Collection of Empty Drug Containers (BIN)

The second author (KS) met with the owners and farm staff 1 day before the start of the observation period. The methodology was explained/reinforced, and labelled plastic bags were provided. Staff were instructed to place discarded packaging (empty or partially full: bottles, boxes of pills/boluses, empty infusion tubes or other) of any drug used on-farm (administered by them or a visiting veterinarian) into the plastic bags throughout the observation period. The farm staff decided where to place the bags to facilitate the collection on their respective farms. Bags were collected 1 day after the end of each observation period. Due to travel logistics, not all farms were visited on the same day or had the same length of the collection period.

Only herd owners who stated “having used drugs” and had items in the bags/bins (*n* = 55 and *n* = 54 herds in sample period 1 and 2, respectively) or those who stated “not having used drugs” and had no items in the bins (*n* = 3 and *n* = 5 herds in sample period 1 and 2, respectively) were considered for each observation period. The contents of the bags from each herd were tallied, and (1) anonymised herd code and collection period, (2) drug commercial name, (3) drug amount and unit, (4) drug concentration (e.g., mg/ml or gr/unit), and (5) name of related active ingredients were recorded into a Microsoft® Excel (Microsoft Corporation, Redmond, WA, USA) spreadsheet for further analysis. If drug packages were not empty, only the amount used was noted. The commercial names were linked to the corresponding code of the Anatomical, Therapeutic, and Chemical Veterinary (ATC_vet_) classification registered under the Swedish Pharmaceutical Industry Association Service (Läkemedelsindustriföreningens Service AB). This is publicly available in the online compendium known as Pharmaceutical specialties in Sweden (www.fass.se). If the commercial name/active ingredients were not found/registered under this system, the ATC_vet_ code was assigned based on information retrieved from the European Head of Medicines Agency (www.hma.eu).

#### Swedish Board of Agriculture Antimicrobial Use Data Extract (SBA)

A data extract of the drugs used by the participating herds comprising the two observation periods was requested from the Swedish Board of Agriculture. Such information is contained in one of the three main DAWA report sections. The reports are submitted by state-employed veterinarians in the form of text files from their computer program LINK, via the DAWA e-service. Most private veterinarians also use a similar format with <30% of them sending in physical practice journal forms.

The original extract had the following information: (1) anonymised herd code, (2) diagnostic code, (3) drug commercial name, (4) ATC_vet_ code, (5) drug unit and amount used, (6) type of treatment (single, group, or all animals), (7) number of animals involved, and (8) treatment date.

The received data extract was screened for usability. The following problems were found, and data records were corrected or discarded as follows. Records with non-existent, incomplete, or incoherent drug name or ATC_vet_ codes were identified. If such records had a recognisable drug name or a partial ATC_vet_ code, a complete code was assigned following the same procedure as for the BIN dataset. Some records had no name or ATC_vet_ code, but instead, a note indicating “error of data transfer,” “drug under license,” or “unknown product” was found. As no valid assumptions could be made about the data entries (i.e., unknown relation), such records were eliminated from further analysis. Lastly, only records/events that took place during the individual herd's observation periods were kept for further investigation.

#### Växa Sverige Data Extract (VXA)

The second data extract of drug use came from Växa Sverige (VXA), the Swedish dairy cattle association that provides on-farm advice and milk recording services to their members. VXA registers 2,003 herds (76% of all Swedish herds) comprising 220,131 animals (78% of all Swedish dairy cows) in their data control system (19, 26). The system captures animal-level events based on their unique national animal identifier. Such events include pedigree, birth/death, cow movements, calving events, milk quantity and quality records, and disease events. An extract of the pedigree, cattle movement, and sickness events was obtained covering the two observation periods of all animals related to the participating herds. Besides, summary statistics per participating herd for the fiscal year October 2016 to September 2017 were also obtained from Växa Sverige.

The pedigree and cattle movement reports are actively updated by on-farm staff, providing information about the actual herd size and composition at the time of the two observation periods. Moreover, the disease reports contained information about drug use at the animal level. Such reports are generated by pulling data from the SBA system, including the drug use, through an active back-end communication interface between the two systems. Additional but minimal input by farm staff can happen, but without affecting drug usage information.

Data extracts were screened for usability and, when necessary, edited. There were no errors found with the pedigree or cow movement data. The animal age and number of days each animal was active in the participating herds at each observation period were calculated. Based on cow age, a new variable was created that classifies animals as either (a) cows/bulls (adult animals >730 days old), (b) heifer/steers (animals >365 ≤730 days old), and(c) young/calves (<365 days old). The common information allowed calculating the individual herds' population at risk of receiving antimicrobial treatment during each observation period so that AMU estimates could be adjusted for herd size and age differences.

Disease data provided the following information at animal level: drug/treatment product as a code, treatment date, diagnosis code, and amount used. The treatment code was cross-matched with a translation code list that provides information about the commercial drug name, and ATC_vet_ code comparable to the SBA dataset. Some data entries matched no name or ATC_vet_ code but had notes indicating “drug under license” or “unknown product.” Such entries (i.e., unknown relation) were removed from further analysis. Lastly, only records/events that mirrored the individual on-farm BIN observation periods were kept for further investigation.

### Estimation of Herd-Level AMU

A full list of commercial drug names and linked ATC_vet_ codes identified across the three datasets was compiled. The list contained only ATC_vet_ codes mentioned in the European Medicines Agency (EMA) protocol (27) for AMU quantification. These ATC_vet_ code groups include (A) Intestinal/oral (O) use: QA07AA, QA07AB; (B) Intrauterine (IU) use: QG01AA, QG01AE, QG01BA, QG01BE, QG51AA, QG51AQ; (C) Systemic/Injectable (IN) use: QJ01; (D) Intramammary tubes (IMt): QJ51; and (E) Antiparasitic agents: QP51AG. The final list had 16 individual ATC_vet_ codes, representing 26 commercial products, of which 38% were products with a combination of drugs (i.e., products had two or more active ingredients in its composition). We used this list to select records relevant for AMU calculations from each dataset and the herd/dataset/period AMU metric calculations.

Animals of all ages were included in the AMU calculations. The standardised live weight used in the EMA protocol is lower than the national average in Swedish national statistics (19, 28). Instead, it was decided to use the following standard weights. Cows/bulls (i.e., adults animals) = 600 kg; heifers/steers (i.e., pubescent animals) = 300 kg; and calves (i.e., young animals) = 100 kg, as these figures represent the Swedish national herd. Production days were defined as the actual number of days an animal was kept in the herd during the observation periods, according to livestock movement data in the VXA dataset extract. The number of cow-years per herd was calculated using the total number of cows' production days per herd divided by 365.

Amounts of active antimicrobial substance were calculated by multiplying the volume administered to the animals (usually in ml) by the concentration of the active antimicrobial substance (e.g., mg/ml) to give the total mass of active antimicrobial substance in mg for each dataset. Then, the number of defined daily doses (DDD_vet_) and defined course doses (DCD_vet_) administered was calculated using the DDD_vet_ and DCD_vet_ values assigned to the individual antimicrobial substances (based on ATC_vet_ code) and animal species by the European Medicines Agency (29, 30). The number of defined daily doses per animal and year (nDDD_vet_/animal/year), the number of defined course doses per animal and year (nDCD_vet_/animal/year) for the individual ATC_vet_ code, and the summation of all codes by herd were calculated following the formula as advised by the network on quantification, benchmarking and reporting of AMU at farm level (AACTING) (13) as follows:

(1) $$\begin{array}{l} \sum_{\text{i}=1}^{\text{n}}\frac{\text{amount }{\text{AI}}_{\text{i}}\text{ in period P }\left(\text{mg}\right)}{{\text{DDDvet}}_{\text{i}}\;\left(\frac{\text{mg}}{\text{kg}/\text{day}}\right)\;\times \text{ \# animal days in period P }\left(\text{days}\right)\times \text{ standard weight }\left(\text{kg}\right)}\;\left(365\text{ days}\right) \end{array}$$

where:

***AI***_***i***_ = amount (in mg) of active ingredient *i* used in period *P*

***i*** = 1, 2, …, n

***# animal days in period P*** = # animals present daily during *P* (days).

***Standard weight*** = standard cow weight at treatment (in kg)

***DDDvet***_***i***_ = Defined Daily Dose of active ingredient i (in mg/kg/day); to calculate the number of days under treatment over the defined period. If ***DDDvet***_***i***_ is replaced by ***DCDvet***_***i***_ (Defined Course Doses), then the average number of courses per animal will be calculated.

***DDDvet***_***i***_ & ***DCDvet***_***i***_ can also be expressed in terms of the number of items (e.g., IMt, bolus or pills), in which case the number of items used in period P will be used in the formula instead of the amount of active ingredient. Lactating intra-mammary tubes are dosed at the number of tubes/cow/day, while dry cow tubes are dosed as “4/cow” as a single treatment, and intrauterine products are one unit per cow. Thus, ***DCDvet***_***i***_ figures can be calculated for dry-cow intra-mammary tubes and intrauterine products.

Similar calculations were done according to the administration route for each drug formulation (i.e., IN, O, lactation IMt, dry-cow IMt, and IU) and by the classification of critically important antimicrobials (CIAs) set by EMA/AMEG/2016 (5, 25, 31) at the time when data was collected. The classification set by EMA is a categorisation of the list of highest-priority CIAs (HP-CIAs) for humans set by the World Health Organization (WHO) (6). The EMA classification aims to consider and advise on the public health risk from the use of antimicrobials in animals expressed by WHO (6, 32), but balancing against the need to protect animal health—providing a One Health context considering the needs of humans, animals, and environment and at the same time these sectors as sources of AMR (5, 31). The EMA/AMEG/2016 classification (5, 25) includes three categories: (1) antimicrobials used in veterinary medicine, where the risk for public health is estimated as low or limited; (2) antimicrobials used in veterinary medicine where the risk for public health is estimated higher; and (3) antimicrobials not approved for use in veterinary medicine.

### Statistical Analyses

All analyses were conducted in SAS/STAT 14.3 (SAS Institute Inc., Cary NC, USA).

#### Descriptive Presentation of Herd Characteristics and AMU Metrics

A descriptive analysis of the key herd characteristics (VXA dataset) and AMU metrics as DDD_vet_ and DCD_vet_/animal/year (all datasets) by active ingredient was done by calculating the mean, median, standard deviation, and interquartile range. Similar descriptive analyses were done for the summation of all ATC_vet_ codes within the herd (i.e., total DDD_vet_/animal/year and total DCD_vet_/animal/year) and the total split by drug administration form or by CIAs.

#### AMU Dataset Agreement and Biases Analyses

Bland–Altman plots consisting of the mean between two datasets [(Dataset A + Dataset B)/2] of the herd total DCD_vet_/animal/year plotted against the difference between the two datasets (Dataset B–Dataset A) were constructed. The mean difference *d* between dataset A and B represents the bias or lack of agreement between datasets. The standard deviation of the difference *d* represents the variability of the differences and is used to calculate 95% limits of agreement between the datasets. The 95% limits of agreement represent the range within which 95% of the observations (i.e., differences between dataset A and B) fall. They are not confidence limits but function instead as a reference interval (33). If the values of the differences within the range are considered “clinically acceptable,” then the two methods could be used interchangeably. The mean bias of the methods and the SD of the bias were calculated, across the three datasets for the herd total nDCD_vet_/animal/year and also split according to the drug administration form. Ninety-five % limits of agreement, calculated as the mean difference in dataset measures ±1.96^*^SD, were calculated and labelled on the Bland–Altman plots. A horizontal line at y = 0 was added to the plot to indicate the line of equality upon which all points would lie if both methods yielded the same results. Plots were then examined visually to identify any patterns in the data. A second plot line was investigated. This corresponded to the potential for bias that is not constant across the range of values (proportional bias). For that, a linear regression model was fitted for each dataset, with the VXA or SBA (Dataset A) dataset as the outcome variable and BIN (Dataset B) dataset as the independent variable. The slope of the regression line was used to evaluate the extent of systematic bias between two particular datasets.

#### Analysis of AMU Differences Between Conventional and Organic Dairy Herds

The association between the production system (conventional vs. organic) and AMU was assessed using linear mixed models (PROC MIXED), with the total number of DCD_vet_/animal/year as the dependent variable and sampling period included as a repeated effect within herd. This procedure was done for BIN and VXA datasets separately. The residuals were tested for normality both visually and analytically; when a variable was not normally distributed, the Box–Cox methodology was used to identify the most appropriate transformation. The analyses were undertaken on the transformed, normally distributed data, and back-transformed results are presented. Fixed effects tested in the model were production system, observation period, drug administration type (IN, lactating IMt, dry-cow IMt, O, and IU), and the interaction between production type and drug administration type. Compound symmetry was the selected covariance structure used for all models. Factors significantly (*P* < 0.05) associated with the dependent variable were retained in the model.

## Results

### Herd Characteristics and the Number of Farms With Evidence of AMU

A summary of participating herds' characteristics according to their production system is presented in Table 1. Organic and conventional farms had a similar herd size. However, organic farms had lower milk production, mortality, lameness, and clinical mastitis caseload, but higher bulk milk somatic cell count than conventional farms.

**Table 1:** Yearly herd characteristics in a sample of 26 organic and 25 conventional Swedish dairy herds.

**Yearly herd characteristics (Reporting period Oct. 2016–Sep. 2017)**	**Production system**	**Mean**	**Lower quartile**	**Median**	**Upper quartile**
Average number cows/year	Organic	103.8	66.3	73.1	133.2
	Conventional	107.7	66.7	101.5	138.2
Energy corrected milk production (*kg/cow/year)*	Organic	9,464	8,763	9,261	10,057
	Conventional	10,545	10,132	10,743	11,055
^*^Bulk milk somatic cell count *(1,000 cells/ml)*	Organic	282.8	234.0	300.5	327.0
	Conventional	226.6	185.0	216.0	267.0
Mastitis *(cases/100 cows/year)*	Organic	6.8	1.5	4.7	9.2
	Conventional	10.1	3.7	9.0	10.6
On-farm mortality (*cases/100 cows/year)*	Organic	4.1	2.8	4.1	5.2
	Conventional	5.3	3.7	5.1	7.4
Lameness *(cases/100 cows/year)*	Organic	2.6	0.0	1.0	4.1
	Conventional	5.2	0.0	2.0	5.2

* *An average estimate derived from the somatic cell count and milk yield of the individual cows at each monthly test-day*.

*At the time of data extraction; full data was available only for 51/60 herds involved in the study*.

The ratio of available data entries in SBA— “related” (i.e., ATC_vet_ code linked to an antimicrobial formulation), “Not related” (i.e., ATC_vet_ code not linked to an antimicrobial formulation), and with “Unknown relation” to AMU were 352 (27%): 839 (65%): 104 (8%) and 386 (24%): 1,130 (70%): 104 (6%) for periods one and two, respectively. As for the VXA dataset, the ratio of available data entries “related,” “Not related,” and with “Unknown relation” to AMU were 385 (27%): 903 (62%): 159 (11%) and 399 (20%): 1,274 (65%): 289 (15%) for periods one and two, respectively.

Corresponding records of AMU across datasets (i.e., at least one entry of AMU per farm/period across all dataset) were found in 77% of the farm/period entries (*n* = 47 farms P1, *n* = 45 farms P2). Only 2.5% of the farm/period entries had a note in the BIN stating no AMU for the given period and no AMU entry in the SBA and VXA datasets. This left 20.5% of the farm/period entries with mismatching records of AMU across datasets (Figure 1).

### Overall AMU Descriptive Statistics by Dataset, Production Type, and Period

**Figure 2:**
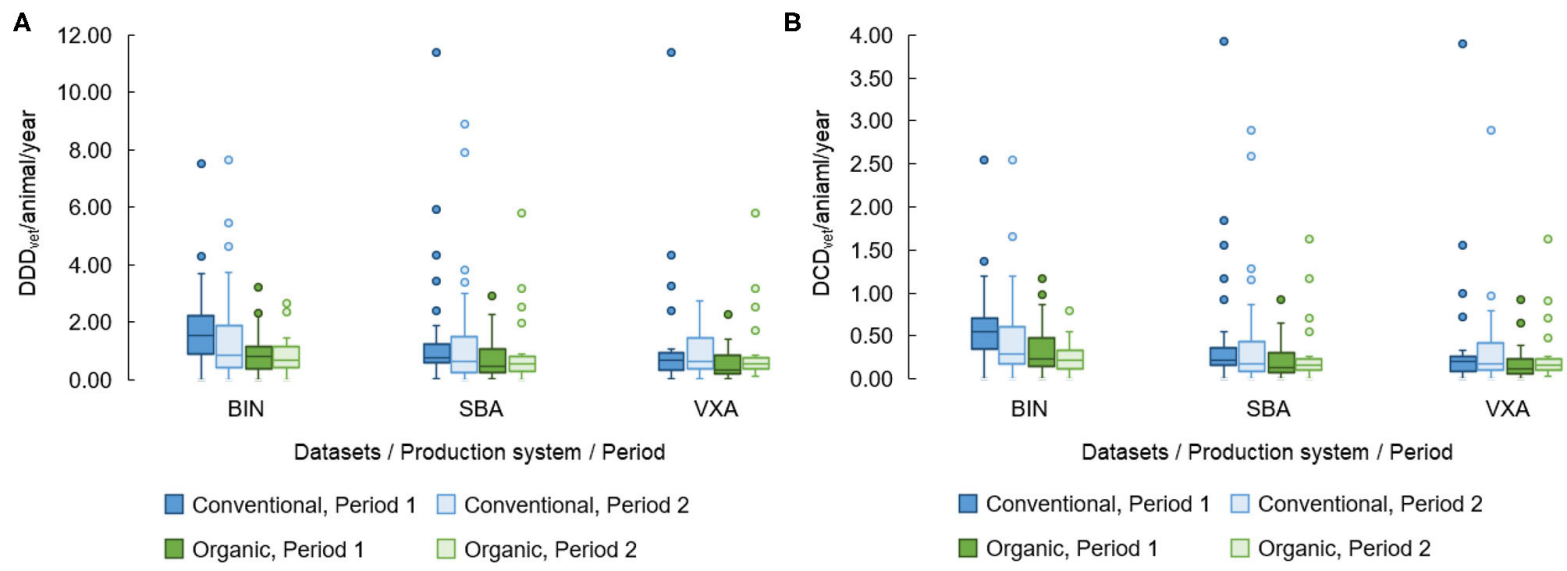
Distribution of **(A)** herd total number of defined daily doses per animal/year (DDD_vet_/animal/year) and **(B)** herd total number of defined course doses (DCD_vet_/animal/year) per animal/year in a sample of organic (*n* = 30) and conventional (*n* = 30) Swedish dairy herds reported across two periods and three datasets. Box = the range between the 1st and 3rd quartiles; horizontal line = median; lower and upper whisker = interquartile range; dots = outliers. BIN, Bin collection method records, *n* = 57 and *n* = 55 herds had reported use in P1 and P2, respectively. SBA, Swedish Board of Agriculture database, *n* = 51 and *n* = 53 herds had reported use in P1 and P2, respectively. VXA, Växa Sverige database; *n* = 48 and *n* = 48 had reported use in P1 and P2, respectively.

The DDD_vet_/animal/year and DCD_vet_/animal/year are presented in Figure 2. At the same time, descriptive statistics are given in the Supplementary Material along with details of AMU concerning ATCvet codes. More herds with confirmed AMU and higher within-herd AMU were observed in the BIN dataset compared to the SBA and VXA datasets. Regardless of dataset, organic herds had a numerically lower total AMU compared to conventional farms.

Across datasets, injectable procaine benzylpenicillin (QJ01CE09) was the main antibiotic prescribed of a list of 16 drug formulations found. For all the drugs used, there was a variation between prescription and the related number of DCDvet/animal/year across datasets for each period/production system. Further details can be found in the Supplementary Material section of this paper.

### Agreement and Biases Between Datasets: Bland–Altman Plots and Regression Analysis Results

The number of herd observations per dataset and the number of corresponding observations between the datasets are shown in Table 2. The number of herds reporting AMU for the primary drug forms (IN and IMt) was higher for the BIN dataset than SBA and VXA. The number of corresponding reports between BIN and the other two datasets was low while between SBA and VXA it was high. The number of corresponding reports between datasets was high for IN formulations but low for IMt formulations, especially for dry-cow IMt. Usage of O drugs was found for two herds. The herds involved and time of observation differed between BIN and the matching reports in the VXA and SBA datasets. No usage of IU drugs was found in the BIN dataset for any herd at any period, but records were found in SBA and VXA for four herds in the first period.

**Table 2:** Slope of the regression line comparing antimicrobial use (AMU) DCD_vet_/animal/year metric of three datasets, and bias, variability of the bias, and limits of agreement for AMU across three datasets in a sample of 60 Swedish dairy herds (30 conventional and 30 organic).

**Drug administration form**	**Comparison** ***A*** vs. B ^*^dataset		**^a^Mean bias**	**SD**	^****b****^**LL and UL of agreement**		**Slope of regression line (Dataset A vs. B)**	***P*-value indicating a significant difference from 1 for the slope**	**Herd × period with at least one record of AMU in Dataset A vs. B, (*n* = concurrent records found, % of total herd observations within category)**
All forms	*BIN*	VXA	−0.15	0.315	−0.766	0.469	0.16	**0.011**	108/96 (92, 77%)
		SBA	−0.08	0.337	−0.738	0.583	0.30	**<0 0.0001**	108/104 (98, 82%)
	*SBA*	VXA	−0.07	0.258	−0.577	0.435	−0.13	**0.003**	104/96 (96, 81%)
Injectables/Parenteral	*BIN*	VXA	−0.11	0.248	−0.598	0.374	0.18	**<0.0001**	107/96 (92, 81%)
		SBA	−0.08	0.239	−0.545	0.394	0.25	**<0.0001**	107/102 (96, 85%)
	*SBA*	VXA	−0.04	0.128	−0.287	0.214	−0.08	**0.017**	102/96 (96, 85%)
Intramammary lactation tubes	*BIN*	VXA	−0.01	0.092	−0.190	0.171	0.12	0.194	38/31 (23, 47%)
		SBA	0.05	0.219	−0.379	0.479	0.73	**<0.0001**	38/38 (27, 55%)
	*SBA*	VXA	−0.06	0.215	−0.481	0.362	−0.60	**<0.0001**	38/31 (31, 63%)
Intramammary dry–cow tubes	*BIN*	VXA	−0.07	0.123	−0.315	0.169	0.06	0.759	54/12 (9, 15%)
		SBA	−0.05	0.137	−0.321	0.215	0.19	0.358	54/21 (14, 23%)
	*SBA*	VXA	−0.02	0.058	−0.134	0.094	−0.08	0.227	21/12 (12, 20%)

a *The mean bias represents the difference between datasets as defined course doses/animal/year “B” —defined course doses/animal/year “A”. ^b^The LL (lower agreement limit) and UL (upper agreement limit) represents the mean “bias” −1.96 × SD and the mean “bias” + 1.96 × SD, respectively*.

* *BIN, Bin collection method records; SBA, Swedish Board of Agriculture database; VXA, Växa Sverige database. Bold values indicate the P-value <0.05*.

The mean differences in the herd total DCD_vet_/animal/year metric between datasets for the different drug forms, i.e., the mean bias, the standard deviations of these differences, and the limits of agreement, are shown in Table 2. Again, a greater AMU was reported in the BIN than in the SBA and VXA datasets (mean bias < 0). However, when comparing BIN and SBA datasets for lactating IMt, higher AMU was reported in the SBA dataset than in the BIN (mean bias > 0).

**Figure 3:**
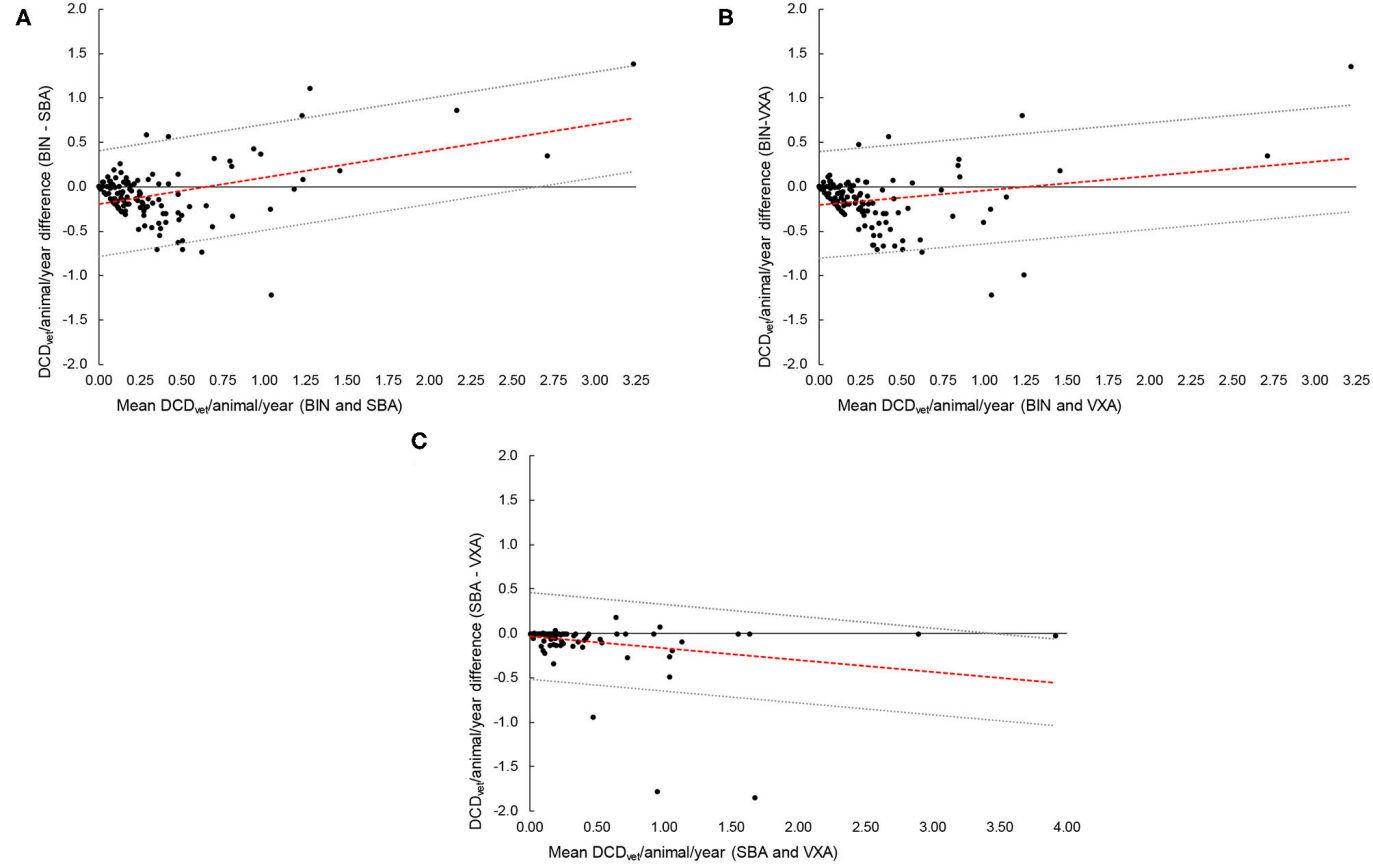
Bland–Altman plots of **(A)** BIN vs. SBA dataset **(B)** BIN vs. VXA dataset and **(C)** SBA vs. VXA datasets on a sample of 60 farms in Sweden. (−−−) Regression line between the measures; (….) Limits of agreement for the regression line; (____) Line of equality (y = 0).

Figures 3A–C present a selected example (all drug types) of AMU metric comparisons between BIN vs. SBA, BIN vs. VXA, and SBA vs. VXA, respectively. Very few data points were found in the line of equality (y = 0), thus confirming discrepancies between datasets. A great variability but no clear pattern was observed between the datasets. The presence and extent of potential systematic bias between compared datasets were evaluated with a regression line (red dotted line in Figure 3) and related agreement limits. If two dataset metrics are similar, then the regression line should be coincident with the line of equality (x = y), i.e., the slope of the regression line should be equal to one. For most of the comparisons made, the slopes were significantly different from one, indicating no real agreement between the methods (Table 3). However, for dry-cow IMt (all comparisons) and lactating IMt (BIN vs. SBA), the slopes of the regression lines were not significantly different from one (Table 2), indicating agreement between the dataset for those farms where IMt was recorded.

**Table 3:** Associations between production type and drug administration form [least-square (LS) means, 95% confidence intervals (CI)] on antimicrobial use (obtained from VXA data) measured as the number of defined courses animal/year (DCD_vet_/animal/year) and as estimated in a linear mixed model, in a sample of 56 (27 Organic and 29 Conventional) Swedish dairy herds.

	**Factor**	^*^DCD_****vet****_**/animal/year**			
		**LS mean**	**Low 95% CI**	**High 95% CI**	***P*-value**
Production type	Organic	0.02	0.007	0.040	0.408
	Conventional	0.02	0.011	0.051	
Drug administration form	Intramammary tube (lactating cow)	0.05	0.032	0.087	**<0.001**
	Intramammary tube (dry-cow)	0.06	0.030	0.123	
	Intrauterine	0.02	0.003	0.058	
	Parenteral/injectable	0.14	0.106	0.184	

* *DCDvet/animal/year (i.e., dependent variable) required a Box–Cox transformation for analysis; back-transformed data are presented. Besides production type, only factors significantly (P < 0.05) associated with the dependent variable were retained in the model and are presented in the table. Bold values indicate the P-value <0.05*.

### Associations Between Production Type, Administration Form, and AMU

The associations between total DCD_vet_/animal/year and production type (conventional vs. organic production), observation period, and drug administration form, as estimated in the linear models, in the VXA dataset are given in Table 3 and the BIN dataset in Table 4.

**Table 4:** Associations between production type and drug administration form [least-square (LS) means, 95% confidence interval (CI)] and antimicrobial use (obtained from BIN data) measured as the number of defined courses animal/year (DCD_vet_/animal/year) and as estimated in a linear mixed model, in a sample of 60 (30 organic and 30 conventional) Swedish dairy herds.

**Factor**			^*^DCD_****vet****_**/animal/year**			***P*-value**
			**LS mean**	**Low 95% CI**	**High 95% CI**	
Production type	Organic		0.08	0.059	0.118	***0.067***
	Conventional		0.13	0.096	0.171	
Drug administration form	Intramammary tube (lactating cow)		0.04	0.030	0.066	**<0.001**
	Intramammary tube (dry-cow)		0.09	0.065	0.119	
	Parenteral/injectable		0.25	0.206	0.304	
^**^Interaction: (Production type) × (Drug administration form)	Intramammary (lactating cow)	Organic	0.03	0.013	0.054	**0.033**
		Conventional	0.07	0.043	0.106	
	Intramammary (dry cow)	Organic	0.09	0.055	0.138	0.970
		Conventional	0.09	0.058	0.129	
	Parenteral/injectable	Organic	0.20	0.148	0.264	**0.026**
		Conventional	0.31	0.240	0.404	

* *DCDvet/animal/year (i.e., dependent variable) required a Box–Cox transformation for analysis; back-transformed data are presented. Besides production type, only factors significantly (P < 0.05) associated with the dependent variable were retained in the model and are presented in the table*.

** *For the interaction, P-values of the pairwise comparison between production systems for each drug administration form are presented. Bold and italic values indicate the P-value <0.05*.

#### VXA Dataset

No difference in AMU was found between production systems and observation period had no association with AMU. However, drug administration form did have an impact on the metric in that injectable drug had the highest AMU metric compared to the other drug presentations, where intrauterine drugs had the lowest AMU metric.

#### BIN Dataset

Numerical but non-statistical differences were found between organic and conventional herds, and no difference in AMU was seen between observation periods. However, drug administration form affected the AMU metric. Injectable drugs had the highest metric while lactating IMt the lowest. An interaction between the production system and drug administration form was found. Injectable drugs and IMt for lactating cows differed by the production system, where organic herds had a lower AMU metric than the conventional herds. However, organic and conventional herds had similar AMU metrics for dry-cow IMt.

## Discussion

To effectively address the situation of AMU and AMR, herd-level data are needed. To our knowledge, this is the first study to provide a full description of AMU regardless of medical indication and split by production type (i.e., organic and conventional herds) in a Swedish dairy context.

The BIN method captured more drug use than SBA and VXA. The first type of discrepancy found was in the number of herds with corresponding reports of AMU across all datasets. The lowest percentage of discrepancies was found between SBA and VXA reports, especially for the injectable forms. Yet, the highest percentage of discrepancies was found between BIN and VXA datasets, especially for intramammary tubes for dry-cow treatments. The second type of discrepancy was in the amount of AMU reported among datasets. Here, Bland–Altman analyses indicated an overall trend of AMU underreporting in the SBA and VXA datasets compared to the reports from the BIN dataset. In most cases, the limits of agreement were large but not beyond what would be considered “clinically acceptable” (33), as in most cases, the disagreement represented <1 treatment course per animal. Consequently, datasets could be used interchangeably. However, as large discrepancies were found for intramammary drug forms, any metrics should take this into account.

A major strength of the BIN dataset is that “overreporting” of AMU is unlikely. This could occur if farm staff discarded outdated or unused drugs, or if half-empty packages/bottles/tubes were reported as fully used. We reduced that risk by making sure that all recorded packages were either empty or reported as the amount used if a half-empty package/bottle was found. In Sweden, the amount of the drug prescribed and dispensed to a farm or individual should match the volume/amount necessary to cover the treatment. Any leftovers must be safely discarded, preventing antimicrobial hoarding or imprudent handling of waste (34). Adherence to this could not be confirmed on the visiting farms. Yet, finding partially used bottles/packages in the BIN might suggest that staff on-farm indeed discard leftovers as required.

However, when SBA or VXA report higher AMU than in the BIN, we have little room to know if actual “underreporting” in the BIN occurred, i.e., if farm staff forgot (intentionally or not) to put the empty packaging in the BIN. In our study, we could establish real, yet probably unintentional, underreporting in the BIN for intrauterine drugs. We found no intrauterine drug packages in the BIN; however, a few such reports were found in the other datasets. Upon discussion with veterinary practitioners, it was understood that the few intrauterine treatments that take place on a farm are performed by the veterinarian on the spot. Hence, the veterinarian routinely discards the gloves, and drug packaging used together without the involvement of the staff on-farm, and the packaging does not reach the bin. This behaviour may also partially explain the overall trend of BIN underreporting AMU for intramammary tubes for lactating cows. These treatments can also be carried out on the spot by the veterinarian, and again, the discarded containers would not necessarily reach the bin.

Several studies have compared AMU based on drug packaging collections (i.e., BIN method) against other data sources, mainly farmers' reports (14, 16, 35). Similar to our study, the authors found that the BIN method/dataset outperforms other data sources. These studies attributed the success of the method to the convenience for staff in reporting AMU with the simple act of discarding packages, saving them from the burden of collating information. In our study, the prescribing veterinarians provided the data for the compared datasets. So, in essence, SBA and VXA reflect prescriptions, while BIN captures actual use. Veterinarians in the research team (KS, NF, and SSL) recognise that AMU reporting is an administrative burden. Depending on the tools available, records can be either collated manually (i.e., pen and paper), scanned and sent to SBA to be uploaded electronically, or transferred manually into a web portal. Alternatively, records could be transferred electronically directly to the SBA database. Transferal could be done on the spot as an individual record or as a bulk of prescriptions later on. Thus, the ability to correctly collate the information is highly depending on the clinical record-keeping. Indeed, upon revision of working datasets ahead of calculations, many treatment records had to be removed as not enough details were present to determine if they were AMU. Poor quality in reporting could explain why some herds failed to present records in SBA or VXA datasets when treatment was performed based on what was found in the BIN dataset. Another explanation could be that the full veterinary prescription was issued before the study period and hence did not appear in the databases. However, the actual use of the drug occurred during the study period, as shown in the BIN.

Nevertheless, the observed discrepancies are consistently larger for intramammary forms, primarily when related to dry-cow treatments (77 to 85% of missing records in either SBA or VXA datasets compared to BIN). Thus, contrary to what is expected, some prescriptions do not get reported as required. In Sweden, current recommendations condemn the storage of antimicrobials (e.g., hoarding of antimicrobial leftovers). Moreover, blanket treatments or preventive AMU is not allowed; selective dry cow therapy is permitted only in individual animals, and cows will only get treated after a diagnosis is made (36, 37). Thus, the lack of reports in SBA and VXA datasets compared to the BIN might be an indication of some practice deviations. If so, further understanding of the quality, magnitude, and drivers of this are needed.

Based on our results, it could be said that BIN provided more information on AMU at herd level for short observation periods. Yet, it is unsustainable for long periods and challenging to scale up on a national basis. On the other hand, our study confirms that SBA and VXA could be used interchangeably. Yet, VXA offers the advantage of being the only standalone dataset for obtaining herd-level AMU metrics in the recommended unit, since time-at-risk can be calculated, over a long time, and scaled to a national level. Nonetheless, underreporting of intramammary drug forms needs to be adequately addressed for SBA and consequently for VXA. Here, we suggest the application of a biannual screening of a random selection of herds using the BIN methodology. This exercise would allow formalizing the monitoring and validation of results captured by SBA and the VXA dataset.

With a focus on BIN reports, we found that the studied farms had an average treatment incidence and average course treatment of 0.43 DDD_vet_/animal/year and 0.22 DCD_vet_/animal/year, respectively. Procaine benzylpenicillin (QJ01CE09) was the preferred (92%) antimicrobial to be used in the reported treatment entries, where more than half (56%) were related to udder problems. This is only the second time in more than 20 years that AMU at herd level is published for dairy cattle in Sweden (38, 39). Direct comparison with these and work in other countries where treatment incidences are reported (23, 40, 41) are constrained mainly by variations in sources of data, calculation methodology, and study design (12).

Despite the challenge of direct comparisons between studies, current AMU in Swedish dairy herds is much lower compared to previous reports for Sweden (6.4 DDDcow/1,000 cow-days for injectable; 3.45 DDDcow/1,000 cow-days intramammary drugs) (38). Equally, it is also low in an international comparison of the median number of doses reported (interquartile range 5.5–13.6 DDDcow/1,000 cow-days) (40). Moreover, results agree with latest reports by EMA (42, 43). Such reports use the “population correction unit” (PCU) based on livestock demographics to estimate the total weight of the livestock population in each country to then compare AMU across the EU in mg/PCU. The report indicates that Sweden is the EU member state with the lowest AMU and after Iceland and Norway the third lowest within all European countries (43).

Only 16 ATC_vet_ codes, representing six antimicrobial classes, were found across the datasets. The list is smaller than that previously reported in Sweden (39, 44) or found in other international reports (40). For example, our study found no reports of macrolides. This use was common 20 years ago and continues to be so in some countries (40). We found no ATC_vet_ codes representing antimicrobial groups like cephalosporins, amphenicols, lincosamides, or pleuromutilins. Injectable (QJ01CE09, 92% farms) and intramammary penicillins (QJ51CE09, 32% farms) were the most used antimicrobials followed by tetracyclines (QJ01AA06, 17% farms), albeit with a low DDD_vet_/animal/year value compared to that of penicillins. Internationally, the most reported antimicrobial groups in dairy cattle are penicillins, and third-generation cephalosporins (40) and highest-priority critically important antibiotic (HPCIA) treatments of mastitis could range from 10 to 80% depending on the veterinary practice (44). In our study, udder health was also the main reason behind the observed AMU. Yet, the HPCIA treatments were low, and cephalosporin use was not recorded at all. HPCIA category 1 includes macrolides, certain penicillins, and tetracyclines, while category 2 includes ampicillins, aminoglycosides, quinolones, 3rd- and 4th-generation cephalosporins, and polymyxins (e.g., colistin).

Moreover, our study also found large variations of HPCIA treatment percentages across farms. Yet, that includes many herds with no HPCIA treatments for category 2. Furthermore, the HPCIA reports for category 2 were mainly due to the use of aminoglycosides or quinolones but not to cephalosporins as in other countries. Sweden has a long-standing history of strengthening its policy recommendations across the human and veterinary sectors to reduce the AMR burden (43, 45). The efforts made have had a definite impact on the reduction as well as the pattern of AMU (17).

Previous studies comparing organic and conventional dairy herds under Swedish conditions found a marginal difference in udder health and reproductive performance, implying equally good animal health in both systems (46–48). Our study presented marginal differences between organic and conventional farms. It should be noted, however, that the small differences are due to an equally low AMU in conventional Swedish farms. Organic herds had a small advantage in that they had a lower mortality and less lameness and mastitis cases. Yet, the bulk milk somatic cell counts were higher for organic farms. Differences had been reported elsewhere between organic and conventional herds (23, 24), indicating that organic herds had a substantially lower level of AMU, but the difference in management between organic and conventional herds is typically larger outside Sweden (49, 50).

Organic herds had numerically lower treatment incidence and a lower number of treatment courses regardless of dataset or treatment form. Yet, at a closer look on the BIN dataset and DCD_vet_/animal/year metric, an interaction between the production system and drug administration form was found. Organic herds had a significant lower AMU for injectables and intramammary lactating cow treatment forms. Yet, organic and conventional farms had a similar AMU for intramammary dry-cow treatment forms.

A potential explanation for the AMU difference between organic and conventional herds for particular drug forms, as suggested by others (23, 24), could be the underlying marginal difference in udder health between production systems in Sweden. Yet, evidence also exists that multiple factors beyond production type characteristics drive the choice of therapy and related management practices, such as farmers' and veterinarians' beliefs and social pressure (51, 52). A qualitative enquiry in the study herds could not relate any particular AMU perception to production type. Still, the authors highlight some behavioural discord among the farmers and veterinarians around udder treatments (53). This, in combination with our findings of underreporting of dry-cow intramammary tubes, suggests that these behaviours are a product of a different set of beliefs around the treatment of cows at dry-off and the practicalities of prescription and administration across herds, but this requires further investigation.

## Conclusion

We conclude that herd-level assessment of AMU is possible using reported prescriptions in combination with herd data, such as in the VXA database, with the caveat that intramammary drugs may be misrepresented. The BIN methodology offered a more comprehensive alternative to gain an account of AMU at herd level. However, as this method is labour- and resource-demanding, it is difficult to apply routinely at a national scale. Thus, for Sweden, we suggest the application of the BIN methodology on a random selection of herds to monitor and validate results captured by SBA and VXA. We also suggest improving the understanding and reduction of underreporting of intramammary drug forms.

This study provides a detailed contemporary account of AMU in Swedish dairy herds. It confirms the reported low AMU based on sales data. Moreover, we conclude that AMU differs between organic and conventional farms for particular drug forms that may partly be driven by the marginal difference in disease prevalence. The use and reporting of dry-cow intramammary drug forms requires further investigation.

## Data Availability Statement

The datasets for this article are not publicly available because of privacy and confidentiality reasons. Requests to access the datasets should be directed to the corresponding author, Jordbruksverket (jordbruksverket@jordbruksverket.se) and Växa Sverige (info@vxa.se), respectively.

## Ethics Statement

Ethical review and approval was not required for the animal study because the study design adhered to the good scientific practice guidelines set out by Swedish legislation and did not include direct animal testing. Thus, no further ethical evaluation or permit was required for its execution. Written informed consent was obtained from the owners for the participation of their animals in this study.

## Author Contributions

GO was in charge of the analysis and wrote the manuscript. KS was in charge of the data acquisition and made a substantial contribution to the conception and revision of the document. SS and NF provided valuable expertise in the conceptualisation of the idea and writing process. UE assisted in the conceptualisation of the idea, analytical process, and revision of the manuscript. All the authors have read and approved the manuscript.

## Conflict of Interest

The authors declare that the research was conducted in the absence of any commercial or financial relationships that could be construed as a potential conflict of interest.
