# Supplementary material for: Antibiotic Use in Organic and Non-organic Swedish Dairy Farms: A Comparison of Three Recording Methods
Source: Front Vet Sci. 2020 Oct 30;7:568881. doi: 10.3389/fvets.2020.568881 (PMC7673384; doi:10.3389/fvets.2020.568881)
Supplement: Supplementary file 1 [file Data_Sheet_1.PDF]

*Supplementary Material*

# Antibiotic Use in Organic and Non-Organic Swedish Dairy Farms: A Comparison of Three Recording Methods

**Gabriela Olmos Antillón*, Karin Sjöström, Nils Fall, Susanna Sternberg Lewerin, Ulf Emanuelson**

*** Correspondence:** gabriela.olmos.antillon@slu.se

**Supplementary Material Section A -** Descriptive statistics of the total number of defined daily doses per animal/year ($DDD_{vet}$/animal/year) and defined course doses per animal/year ($DCD_{vet}$/animal/year), for a sample of 30 organic and 30 conventional Swedish dairy herds across two observation periods and as reported by three different datasets.

| Production type | *Dataset | Period 1: February 2016 – May 2016 x̄, min-max days per herd = 110, 96 – 119 | | | | Period 2: November 2016 – March 2017 x̄, min-max days per herd = 150, 101 – 150 | | | |
|---|---|---|---|---|---|---|---|---|---|
| | | **n= | $DDD_{vet}$/animal/year | | | **n= | $DDD_{vet}$/animal/year | | |
| | | | Mean | Min | Max | | Mean | Min | Max |
| Organic | BIN | 27 | 0.94 | <0.0001 | 3.214 | 28 | 0.84 | <0.0001 | 2.666 |
| | SBA | 24 | 0.76 | 0.004 | 2.886 | 25 | 0.92 | <0.0001 | 5.772 |
| | VXA | 23 | 0.62 | 0.004 | 2.274 | 23 | 0.96 | 0.099 | 5.772 |
| Conventional | BIN | 30 | 1.77 | <0.0001 | 7.528 | 27 | 1.62 | <0.0001 | 7.633 |
| | SBA | 27 | 1.57 | 0.002 | 11.382 | 28 | 1.42 | <0.0001 | 8.871 |
| | VXA | 25 | 1.37 | 0.002 | 11.382 | 25 | 1.18 | 0.009 | 8.871 |
| **Production type** | ***Dataset** | **n= | $DCD_{vet}$/animal/year | | | **n= | $DCD_{vet}$/animal/year | | |
| | | | Mean | Min | Max | | Mean | Min | Max |
| Organic | BIN | 27 | 0.34 | <0.0001 | 1.166 | 28 | 0.27 | <0.0001 | 0.828 |
| | SBA | 24 | 0.23 | 0.001 | 0.938 | 25 | 0.30 | <0.0001 | 1.631 |
| | VXA | 23 | 0.19 | 0.001 | 0.918 | 23 | 0.29 | 0.032 | 1.631 |
| Conventional | BIN | 30 | 0.60 | <0.0001 | 2.543 | 27 | 0.52 | <0.0001 | 2.540 |
| | SBA | 27 | 0.51 | <0.0001 | 3.923 | 28 | 0.46 | 0.002 | 2.891 |
| | VXA | 25 | 0.45 | <0.0001 | 3.897 | 25 | 0.37 | 0.002 | 2.891 |

*BIN= Bin collection method records; SBA= Swedish Board of Agriculture database; VXA = Växa Sverige database.
**n= indicated the number of herds that had reported antimicrobial use within the specified production type, dataset and time period

**Supplementary Material Section B -** Descriptive statistic of defined daily doses per animal/year and defined course doses per animal/year for different antibiotic forms (Table A, B1, B2, C1 and C2)

## 1 Supplementary Table A. Descriptive statistic of defined daily doses per animal/year ($DDD_{vet}$/animal/year) and defined course doses per animal/year ($DCD_{vet}$/animal/year), for **oral and intrauterine antibiotic forms** as classified by their $ATC_{vet}$ code used in a sample of 60 Swedish dairy herds across two observation periods as reported by three different datasets.

| $ATC_{vet}$ Code | Antibiotic class | Dataset | Period 1: February to May 2016<br>x̄, min-max days per herd = 110, 96 – 119<br>Herds with reported use (n=, % of 60) | | $DDD_{vet}$/animal/year<br>mean, median (min, max) | $DCD_{vet}$/animal/year<br>mean, median (min, max) | Period 2: November 2016 – March 2017<br>x̄, min-max days per herd = 150, 101 – 150<br>Herds with reported use (n=, % of 60) | | $DDD_{vet}$/animal/year<br>mean, median (min, max) | $DCD_{vet}$/animal/year<br>mean, median (min, max) |
|---|---|---|---|---|---|---|---|---|---|---|
| QA07AA90 | Aminoglycocide | BIN | 0 | 0 | - | - | 1 | 1.7 | 0.003 | 0.001 |
| | | SBA | 1 | 2 | 0.0007 | 0.0003 | 0 | 0 | - | - |
| | | VXA | 1 | 1.7 | 0.0007 | 0.0003 | 0 | 0 | - | - |
| QG01AA07 | Tetracyclines | BIN | 0 | 0 | - | - | 0 | 0 | - | - |
| | | SBA | 4 | 6.7 | 0.056 0.083<br>0.0218 0.1955 | 0.028 0.041<br>0.0109 0.0977 | 0 | 0 | - | - |
| | | VXA | 4 | 6.7 | 0.066 0.088<br>0.0239 0.1955 | 0.033 0.044<br>0.0119 0.0977 | 0 | 0 | - | - |

2 **Supplementary Table B1.** Descriptive statistic of defined daily doses per animal/year ($DDD_{vet}$/animal/year) and defined course doses per animal/year ($DCD_{vet}$/animal/year), for all **injectable antibiotic drugs with a single active ingredient** classified by their $ATC_{vet}$ code used in a sample of 60 Swedish dairy herds across two observation periods as reported by three different datasets.

| $ATC_{vet}$ Code | Antibiotic class | Dataset | Period 1: February to May 2016<br>x̄, min-max days per herd = 110, 96 – 119 | | | | | | Period 2: November 2016 – March 2017<br>x̄, min-max days per herd = 150, 101 – 150 | | | | | |
|---|---|---|---|---|---|---|---|---|---|---|---|---|---|---|
| | | | Herds with reported use (n=, % of 60) | | $DDD_{vet}$/animal/year<br>mean, median (min, max) | | $DCD_{vet}$/animal/year<br>mean, median (min, max) | | Herds with reported use (n=, % of 60) | | $DDD_{vet}$/animal/year<br>mean, median (min, max) | | $DCD_{vet}$/animal/year<br>mean, median (min, max) | |
| QJ01AA06 | Tetracyclines | BIN | 5 | 8.3 | 0.079 | 0.102 | 0.022 | 0.029 | 10 | 16.7 | 0.054 | 0.090 | 0.015 | 0.025 |
| | | | | | 0.0338 | 0.1972 | 0.0095 | 0.0557 | | | 0.0251 | 0.3596 | 0.0071 | 0.1016 |
| | | SBA | 5 | 8.3 | 0.075 | 0.133 | 0.021 | 0.032 | 6 | 10 | 0.087 | 0.091 | 0.025 | 0.026 |
| | | | | | 0.0145 | 0.2953 | 0.0041 | 0.0666 | | | 0.0006 | 0.1798 | 0.0002 | 0.0508 |
| | | VXA | 3 | 5 | 0.044 | 0.045 | 0.013 | 0.013 | 4 | 6.7 | 0.143 | 0.126 | 0.040 | 0.036 |
| | | | | | 0.0145 | 0.0746 | 0.0041 | 0.0211 | | | 0.0392 | 0.1798 | 0.0111 | 0.0508 |
| QJ01CA04 | Penicillins | BIN | 3 | 5 | 0.169 | 0.181 | 0.048 | 0.052 | 3 | 5 | 0.080 | 0.268 | 0.023 | 0.077 |
| | | | | | 0.1390 | 0.2350 | 0.0398 | 0.0673 | | | 0.0411 | 0.6817 | 0.0118 | 0.1951 |
| | | SBA | 2 | 3.3 | 0.009 | 0.009 | 0.003 | 0.003 | 2 | 3.3 | 0.111 | 0.111 | 0.032 | 0.032 |
| | | | | | 0.0042 | 0.0139 | 0.0012 | 0.0040 | | | 0.0513 | 0.1704 | 0.0147 | 0.0488 |
| | | VXA | 2 | 3.3 | 0.009 | 0.009 | 0.003 | 0.003 | 2 | 3.3 | 0.111 | 0.111 | 0.032 | 0.032 |
| | | | | | 0.0042 | 0.0139 | 0.0012 | 0.0040 | | | 0.0513 | 0.1704 | 0.0147 | 0.0488 |
| QJ01CE01 | | BIN | 0 | 0 | - | | - | | 4 | 6.7 | 0.411 | 0.407 | 0.106 | 0.106 |
| | | | | | | | | | | | 0.1427 | 0.6643 | 0.0370 | 0.1722 |
| | | SBA | 0 | 0 | - | | - | | 4 | 6.7 | 0.092 | 0.115 | 0.024 | 0.030 |
| | | | | | | | | | | | 0.0403 | 0.2349 | 0.0104 | 0.0609 |
| | | VXA | 0 | 0 | - | | - | | 4 | 6.7 | 0.092 | 0.115 | 0.024 | 0.030 |
| | | | | | | | | | | | 0.0403 | 0.2349 | 0.0104 | 0.0609 |
| QJ01CE09 | | BIN | 55 | 91.7 | 1.026 | 1.244 | 0.290 | 0.352 | 50 | 83.3 | 0.702 | 1.113 | 0.198 | 0.315 |
| | | | | | 0.0504 | 5.7746 | 0.0143 | 1.6319 | | | 0.0427 | 5.8918 | 0.0121 | 1.6651 |
| | | SBA | 49 | 81.7 | 0.560 | 0.925 | 0.158 | 0.261 | 50 | 83.3 | 0.529 | 1.063 | 0.150 | 0.300 |
| | | | | | 0.0015 | 8.6443 | 0.0004 | 2.4430 | | | 0.0085 | 7.1053 | 0.0024 | 2.0080 |
| | | VXA | 45 | 75 | 0.492 | 0.906 | 0.139 | 0.256 | 48 | 80 | 0.460 | 0.918 | 0.130 | 0.260 |
| | | | | | 0.0015 | 8.6443 | 0.0004 | 2.4430 | | | 0.0085 | 7.1053 | 0.0024 | 2.0080 |
| QJ01MA90 | Quinolone | BIN | 6 | 10 | 0.038 | 0.042 | 0.010 | 0.011 | 3 | 5 | 0.047 | 0.047 | 0.012 | 0.012 |
| | | | | | 0.0228 | 0.0763 | 0.0060 | 0.0200 | | | 0.0400 | 0.0546 | 0.0105 | 0.0143 |
| | | SBA | 5 | 8.3 | 0.047 | 0.158 | 0.012 | 0.042 | 2 | 3.3 | 0.037 | 0.037 | 0.00971 | 0.00971 |
| | | | | | 0.0014 | 0.6554 | 0.0004 | 0.1721 | | | 0.0195 | 0.0546 | 0.0051 | 0.0143 |
| | | VXA | 2 | 3.3 | 0.061 | 0.061 | 0.016 | 0.016 | 2 | 3.3 | 0.037 | 0.037 | 0.00971 | 0.00971 |
| | | | | | 0.0473 | 0.0741 | 0.0124 | 0.0194 | | | 0.0195 | 0.0546 | 0.0051 | 0.0143 |

Supplementary Table B2. **Descriptive statistic of defined daily doses per animal/year ($DDD_{vet}$/animal/year) and defined course doses per animal/year ($DCD_{vet}$/animal/year), for all** injectable antibiotic drugs with a combination of active ingredients **classified by their $ATC_{vet}$ code used in a sample of 60 Swedish dairy herds across two observation periods as reported by three different datasets.**

| $ATC_{vet}$ Code | Antibiotic class | Dataset | Herds with reported use (n=, % of 60) | | Period 1: February to May 2016 x̄, min-max days per herd = 110, 96 – 119 $DDD_{vet}$/animal/year mean, median (min, max) | Period 1: $DCD_{vet}$/animal/year mean, median (min, max) | Herds with reported use (n=, % of 60) | | Period 2: November 2016 – March 2017 x̄, min-max days per herd = 150, 101 – 150 $DDD_{vet}$/animal/year mean, median (min, max) | Period 2: $DCD_{vet}$/animal/year mean, median (min, max) |
|---|---|---|---|---|---|---|---|---|---|---|
| **QJ01EW10** Drug A. Sulfadiazine Drug B. Trimethoprim | **Sulfonamides** | **BIN.a** | 4 | 6.7 | 0.137, 0.158 (0.0588, 0.3001) | 0.050, 0.057 (0.0212, 0.1084) | 1 | 1.7 | 0.086 | 0.031 |
| | | **BIN.b** | | | 0.127, 0.147 (0.0546, 0.2786) | 0.036, 0.041 (0.0153, 0.0780) | | | 0.080 | 0.022 |
| | | **SBA.a** | 2 | 3.3 | 0.049, 0.049 (0.0061, 0.0921) | 0.018, 0.018 (0.0022, 0.0333) | 2 | 3.3 | 0.007, 0.007 (0.0013, 0.0120) | 0.002, 0.002 (0.0005, 0.0043) |
| | | **SBA.b** | | | 0.046, 0.046 (0.0057, 0.0855) | 0.013, 0.013 (0.0016, 0.0239) | | | 0.006, 0.006 (0.0012, 0.0111) | 0.002, 0.002 (0.0004, 0.0031) |
| | | **VXA.a** | 2 | 3.3 | 0.049, 0.049 (0.0061, 0.0921) | 0.018, 0.018 (0.0022, 0.0333) | 1 | 1.7 | 0.012 | 0.004 |
| | | **VXA.b** | | | 0.046, 0.046 (0.0057, 0.0855) | 0.013, 0.013 (0.0016, 0.0239) | | | 0.011 | 0.003 |
| **QJ01EW13** Drug A: Sulfadoxine Drug B. Trimethoprim | | **BIN.a** | 4 | 6.7 | 0.074, 0.087 (0.0273, 0.1712) | 0.020, 0.024 (0.0075, 0.0470) | 4 | 6.7 | 0.060, 0.084 (0.0294, 0.1853) | 0.017, 0.023 (0.0081, 0.0509) |
| | | **BIN.b** | | | 0.074, 0.087 (0.0273, 0.1712) | 0.021, 0.024 (0.0077, 0.0479) | | | 0.060, 0.084 (0.0294, 0.1853) | 0.017, 0.023 (0.0082, 0.0519) |
| | | **SBA.a** | 2 | 3.3 | 0.093, 0.093 (0.0143, 0.1712) | 0.025, 0.025 (0.0039, 0.0470) | 4 | 6.7 | 0.031, 0.044 (0.0008, 0.1113) | 0.009, 0.012 (0.0002, 0.0306) |
| | | **SBA.b** | | | 0.093, 0.093 (0.0143, 0.1712) | 0.026, 0.026 (0.0040, 0.0479) | | | 0.031, 0.044 (0.0008, 0.1113) | 0.009, 0.012 (0.0002, 0.0312) |
| | | **VXA.a** | 2 | 3.3 | 0.093, 0.093 (0.0143, 0.1712) | 0.025, 0.025 (0.0039, 0.0470) | 4 | 6.7 | 0.031, 0.044 (0.0008, 0.1113) | 0.009, 0.012 (0.0002, 0.0306) |
| | | **VXA.b** | | | 0.093, 0.093 (0.0143, 0.1712) | 0.026, 0.026 (0.0040, 0.0479) | | | 0.031, 0.044 (0.0008, 0.1113) | 0.009, 0.012 (0.0002, 0.0312) |
| **QJ01RA01** Drug A. Procaine Benzylpenicillin Drug B. Dihydrostreptomycin | **Penicillin & aminoglycocide** | **BIN.a** | 2 | 3.3 | 0.114, 0.114 (0.0795, 0.1489) | 0.032, 0.032 (0.0225, 0.0421) | 1 | 1.7 | 0.033 | 0.009 |
| | | **BIN.b** | | | 0.050, 0.050 (0.0331, 0.0664) | 0.022, 0.022 (0.0148, 0.0296) | | | 0.017 | 0.008 |
| | | **SBA.a** | 1 | 1.7 | 0.040 | 0.011 | 0 | 0 | - | - |
| | | **SBA.b** | | | 0.017 | 0.007 | | | - | - |
| | | **VXA.a** | 1 | 1.7 | 0.023 | 0.006 | 0 | 0 | - | - |
| | | **VXA.b** | | | 0.009 | 0.004 | | | - | - |

3 **Supplementary Table C1.** Descriptive statistic of defined daily doses per animal/year ($DDD_{vet}$/animal/year) and defined course doses per animal/year ($DCD_{vet}$/animal/year), for all **lactating cows intramammary antibiotic drugs** classified by their $ATC_{vet}$ code used in a sample of 60 Swedish dairy herds across two observation periods as reported by three different datasets.

| $ATC_{vet}$ Code | Antibiotic class | Dataset | Period 1: February to May 2016<br>$\bar{x}$, min-max days per herd = 110, 96 – 119 | | | | | | Period 2: November 2016 – March 2017<br>$\bar{x}$, min-max days per herd = 150, 101 – 150 | | | | | |
|---|---|---|---|---|---|---|---|---|---|---|---|---|---|---|
| | | | Herds with reported use (n=, % of 60) | | $DDD_{vet}$/animal/year<br>mean, median (min, max) | | $DCD_{vet}$/animal/year<br>mean, median (min, max) | | Herds with reported use (n=, % of 60) | | $DDD_{vet}$/animal/year<br>mean, median (min, max) | | $DCD_{vet}$/animal/year<br>mean, median (min, max) | |
| QJ51CE09 | Penicillins | BIN | 19 | 31.7 | 0.119<br>0.0176 | 0.323<br>1.6026 | 0.040<br>0.0059 | 0.108<br>0.5342 | 17 | 28.3 | 0.162<br>0.0086 | 0.378<br>1.7414 | 0.054<br>0.0029 | 0.126<br>0.5805 |
| | | SBA | 19 | 31.7 | 0.191<br>0.0104 | 0.672<br>3.3652 | 0.064<br>0.0035 | 0.224<br>1.1217 | 17 | 28.3 | 0.200<br>0.0003 | 0.461<br>2.6414 | 0.067<br>0.0001 | 0.154<br>0.8805 |
| | | VXA | 13 | 21.7 | 0.197<br>0.0703 | 0.475<br>2.6905 | 0.066<br>0.0235 | 0.158<br>0.8968 | 15 | 25 | 0.203<br>0.0003 | 0.372<br>1.7659 | 0.068<br>0.0001 | 0.124<br>0.5886 |
| QJ51RC23 | Penicillin - combinations | BIN | 3 | 5 | 0.103<br>0.0627 | 0.124<br>0.2065 | 0.034<br>0.0209 | 0.041<br>0.0688 | 2 | 3.3 | 0.296<br>0.1815 | 0.296<br>0.4113 | 0.099<br>0.0605 | 0.099<br>0.1371 |
| | | SBA | 2 | 3.3 | 0.120<br>0.1147 | 0.120<br>0.1253 | 0.040<br>0.0382 | 0.040<br>0.0418 | 2 | 3.3 | 0.144<br>0.0640 | 0.144<br>0.2243 | 0.048<br>0.0213 | 0.048<br>0.0748 |
| | | VXA | 2 | 3.3 | 0.186<br>0.1835 | 0.186<br>0.1880 | 0.062<br>0.0612 | 0.062<br>0.0627 | 2 | 3.3 | 0.107<br>0.0640 | 0.107<br>0.1495 | 0.036<br>0.0213 | 0.036<br>0.0499 |
| QJ51RE01 | Sulfonamides | BIN | 1 | 1.7 | 0.039 | | 0.013 | | 1 | 1.7 | 0.130 | | 0.043 | |
| | | SBA | 0 | 0 | - | | - | | 0 | 0 | - | | - | |
| | | VXA | 0 | 0 | - | | - | | 0 | 0 | - | | - | |

4 **Supplementary Table C2.** Descriptive statistic of defined daily doses per animal/year ($DDD_{vet}$/animal/year) and defined course doses per animal/year ($DCD_{vet}$/animal/year), for all **dry-cow intramammary antibiotic drugs** classified by their $ATC_{vet}$ code used in a sample of 60 Swedish dairy herds across two observation periods as reported by three different datasets.

| $ATC_{vet}$ Code | Antibiotic class | Dataset | Period 1: February to May 2016<br>$\bar{x}$, min-max days per herd = 110, 96 – 119<br>Herds with reported use (n=, % of 60) | | $DDD_{vet}$/animal/year mean, median (min, max) | $DCD_{vet}$/animal/year mean, median (min, max) | Period 2: November 2016 – March 2017<br>$\bar{x}$, min-max days per herd = 150, 101 – 150<br>Herds with reported use (n=, % of 60) | | $DDD_{vet}$/animal/year mean, median (min, max) | $DCD_{vet}$/animal/year mean, median (min, max) |
|---|---|---|---|---|---|---|---|---|---|---|
| QJ51CF02 | Penicillins | BIN | 1 | 1.7 | | 0.006 | 1 | 1.7 | | 0.028 |
| | | SBA | 0 | 0 | NA | - | 0 | 0 | NA | - |
| | | VXA | 0 | 0 | | - | 0 | 0 | | - |
| QJ51RC24 | Penicillins - combination | BIN | 30 | 50 | | 0.114, 0.147 (0.0167, 0.4727) | 22 | 36.7 | | 0.112, 0.110 (0.0182, 0.2943) |
| | | SBA | 7 | 11.7 | | 0.235, 0.223 (0.0223, 0.5710) | 13 | 21.7 | | 0.187, 0.168 (0.0143, 0.3222) |
| | | VXA | 6 | 10 | NA | 0.274, 0.259 (0.0249, 0.5452) | 6 | 10 | NA | 0.167, 0.175 (0.0491, 0.3000) |
| QJ51RC25 | | BIN | 1 | 1.7 | | 0.136 | 2 | 3.3 | | 0.027, 0.027 (0.0090, 0.0454) |
| | | SBA | 0 | 0 | | - | 1 | 1.7 | | 0.076 |
| | | VXA | 0 | 0 | | - | 0 | 0 | | - |

**Table notes applicable to Tables A, B1&2 and C1&2 -** BIN= Bin collection method records, n=57 and n=55 herds had reported use in P1 and P2, respectively. SBA= Swedish Board of Agriculture database, n=51 and n=53 herds had reported use in P1 and P2 respectively. VXA = Växa Sverige database; n=48 and n=48 had reported use in P1 and P2, respectively. Reported use for BIN = at least one container found in the bin. Reported use for SBA and VXA = at least one record associated with the use of antibiotics found in the dataset for the extracted period. The standardised European Medicines Agency $DDD_{vet}$ and $DCD_{vet}$ doses for cows are used for the calculations. Antibiotic categorisation follows the Anatomical Therapeutic Chemical (ATC) Veterinary (VET) Classification System.

**Supplementary Material Section C** - Relative proportion of prescription-entries and the relative proportion of the total DCDvet/animal/year for the different antimicrobials as classified by their ATCvet code split by period/production system are reported in Supplementary Figure 1A and 1B, respectively. The median and range of DDDvet/animal/year and DCDvet/cow/year by ATCvet code/period/dataset is described in supplementary tables (See Supplemental Material) to this paper.

Across datasets, injectable procaine benzylpenicillin (QJ01CE09) was the main antibiotic prescribed of a list of 16 drugs formulations found. For all the drugs used, there was a variation between prescription and the related number of DCDvet/animal/year across datasets for each period/production system. For example, Procaine Benzylpenicillin represented 37%, 46% and 52% of the conventional herds' prescriptions in P1 in the BIN, SBA and VXA datasets, respectively. For the same production type/period procaine benzylpenicillin represented 74%, 63% and 70% of the total DCDvet/animal/year for BIN, SBA and VXA datasets, respectively. Of the total treatment entries described in the SBA dataset, 78% relates to parenteral or injectable forms and were prescribed mainly for udder (56%), musculoskeletal (12%) or systemic [i.e. not associated with a particular body system (10%)] problems (see Supplementary Figure 2).

Across datasets and production systems, only 11.4% of the herd's total number $DCD_{vet}$/animal/year included HPCIAs-Cat 2 (antimicrobials where the risk for public health is estimated higher) [5]. However, the percentage of HPCIAs-Cat2 reported in the BIN dataset was higher (16%) than reported in the SBA (10%) and VXA (8%) (Supplementary Figure 3). The herd median $DCD_{vet}$/animal/year for HPCIAs-Cat2 for the BIN dataset was of 0.070 (min-max = 0 – 0.473) and 0.091 (min-max = 0 – 0.330) in organic and conventional farms, respectively. For 40% of organic herds and 26% of the conventional farms, there was no evidence of the use of HPCIAs-Cat 2 antimicrobials in the BIN.

HPCIA (Highest priority critically important antibiotic)

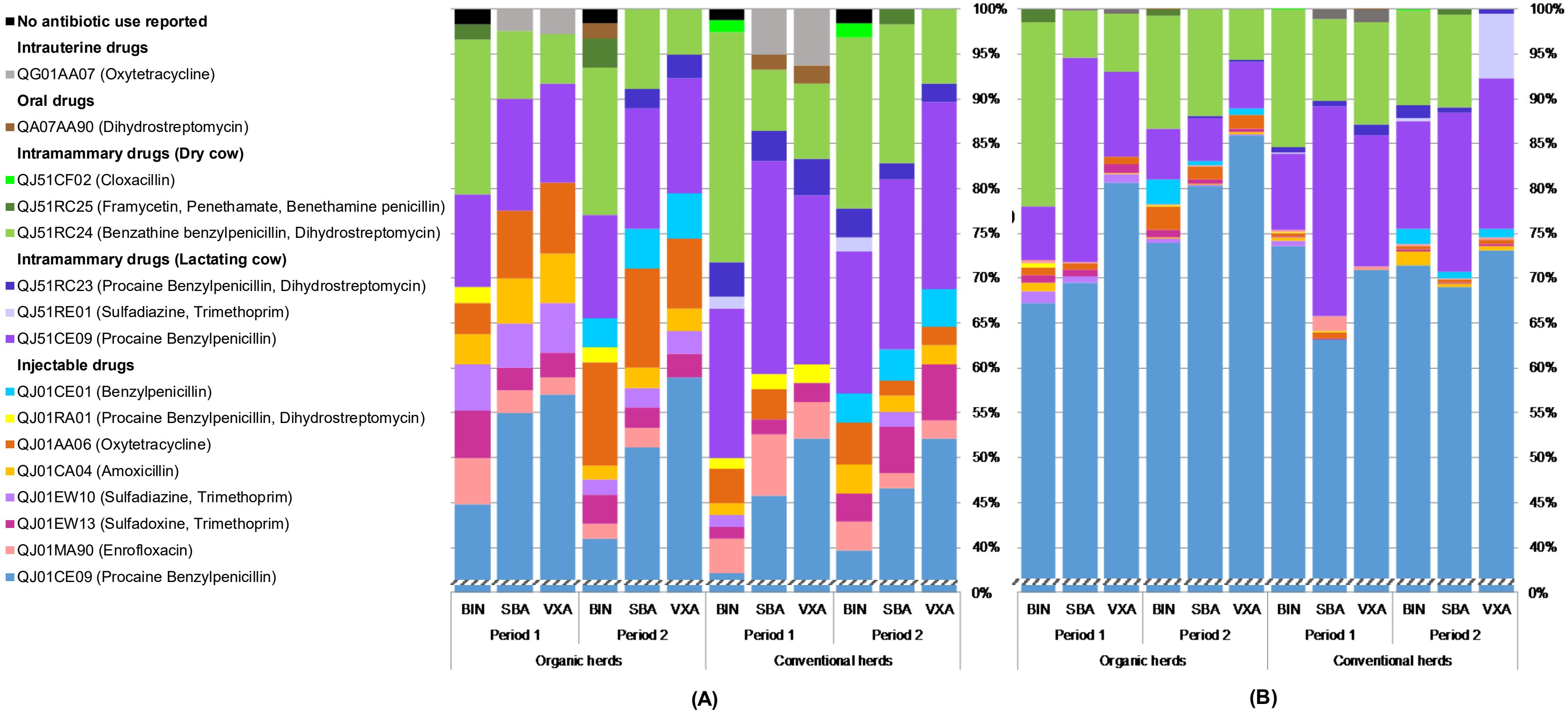


**Supplementary Figure 1. (A)** Proportion of prescription-entries for different classes of antibiotics and **(B)** proportion of total DCD$_{vet}$/animal/year for different antibiotics as classified by their ATC$_{vet}$ code split by two observation periods as reported by three datasets in a sample of 60 (30 organic and 30 conventional) Swedish dairy herds. BIN= Bin collection method records, n=57 and n=55 herds had reported use in P1 and P2 respectively / SBA= Swedish Board of Agriculture database, n=51 and n=53 herds had reported use in P1 and P2 respectively / VXA = Växa Sverige database; n=48 and n=48 had reported use in P1 and P2 respectively.

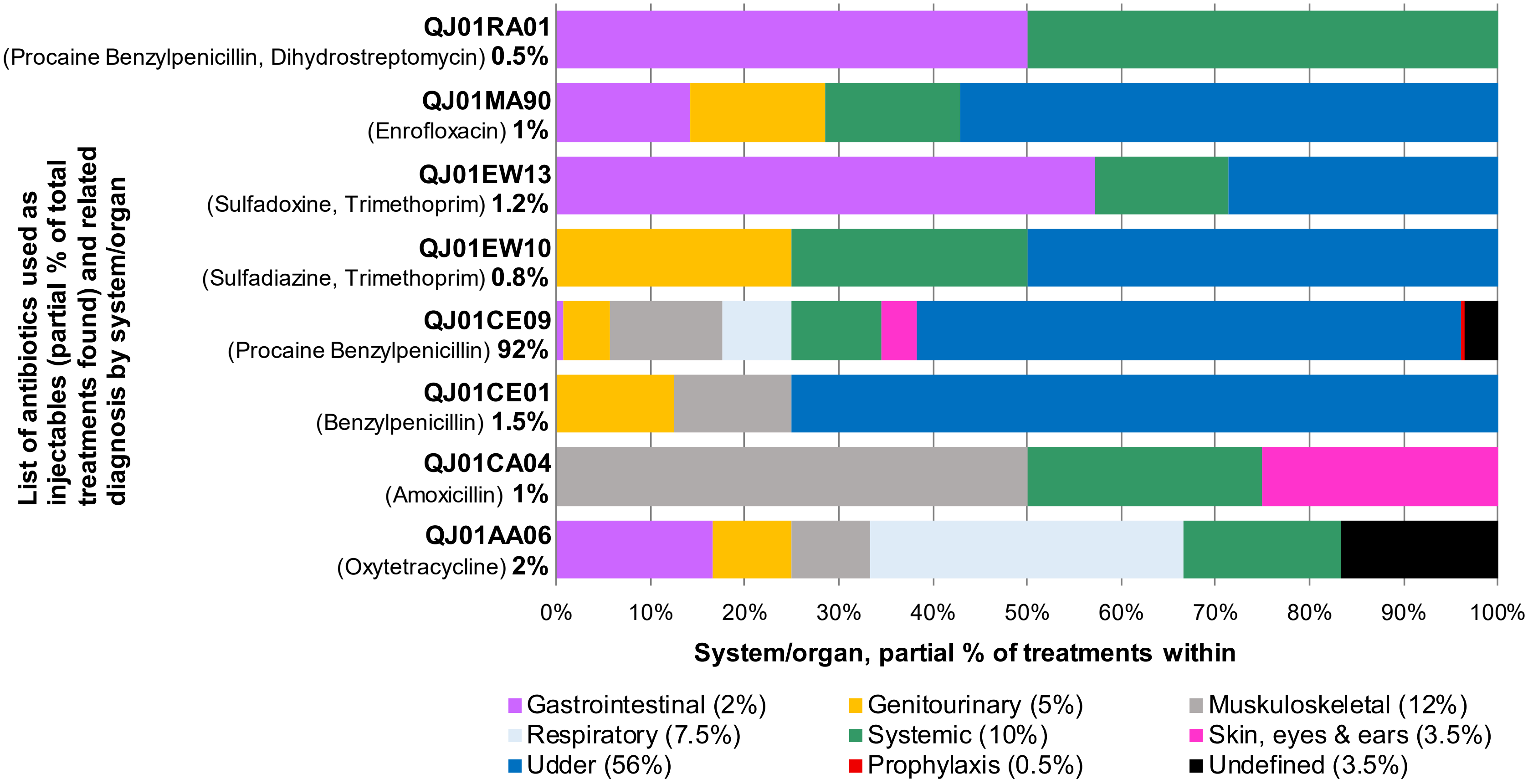


**Supplementary Figure 2.** Proportion of prescription entries (n=557 entries) of injectable antibiotics and related diagnosis classified by organ/system as found in a sample of 60 Swedish dairy farms across two observation periods as reported in the Swedish Board of Agriculture database (SBA). Antimicrobial use records were available for 58/60 farms sampled.

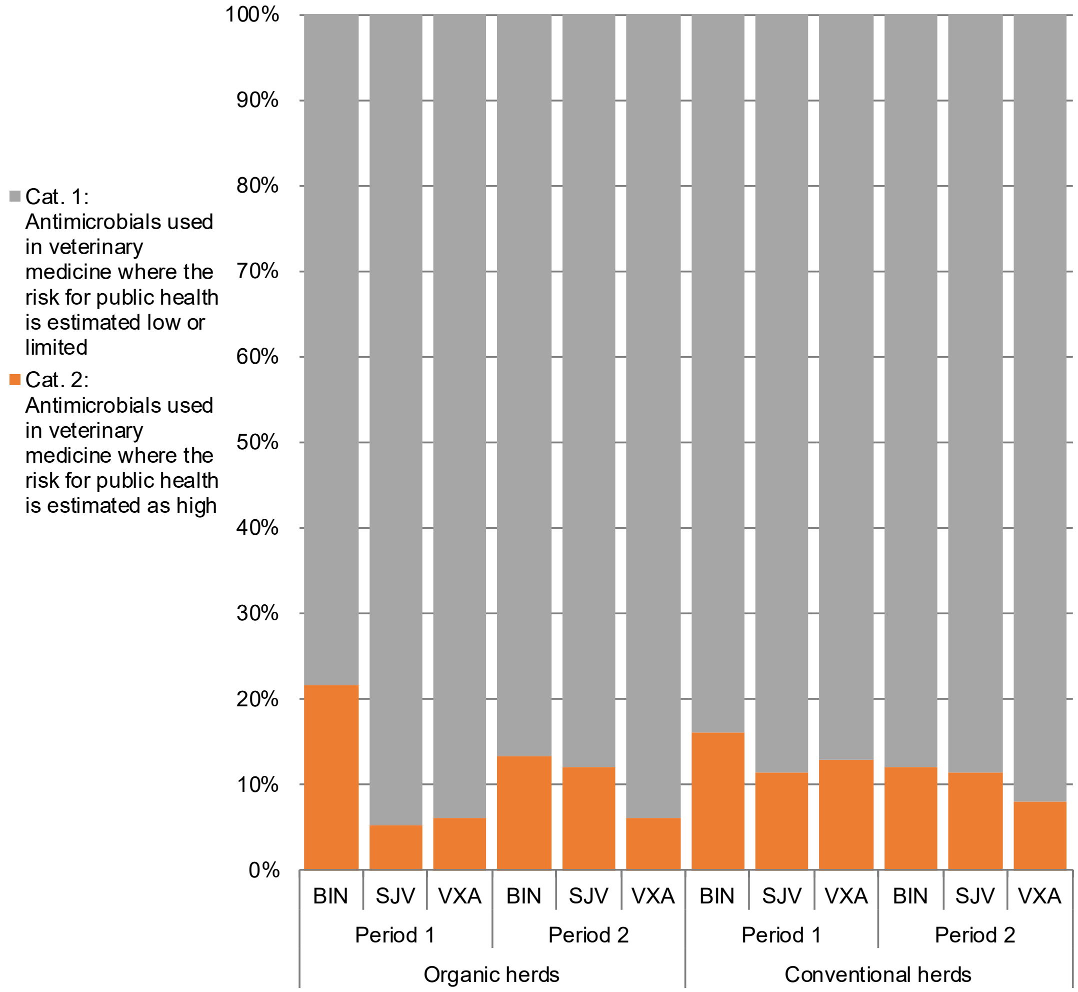


**Supplementary Figure 3.** Proportion of total $DCD_{vet}$/animal/year over two observations periods and production system type as reported by three datasets (BIN= Bin collection method records; SBA= Swedish Board of Agriculture database; VXA = Växa Sverige database) in a sample of 30 organic and 30 conventional Swedish dairy herds. $DCD_{vet}$/animal/year proportions are categorised according to the group of highest priority critically important antimicrobials (HPCIAs) set by the European Medical Agency (EMA\AMEG\2016).
